# Factors associated with self-care behavior in patients with pre-dialysis or dialysis-dependent chronic kidney disease

**DOI:** 10.1371/journal.pone.0274454

**Published:** 2022-10-13

**Authors:** Jung-Won Ahn, Sun Mi Lee, Yon Hee Seo

**Affiliations:** 1 Department of Nursing, Gangneung-Wonju National University, Wonju-si, Gangwon-do, Korea; 2 Department of Nursing Science, Pai Chai University, Seo-gu, Daejeon Metropolitan City, Korea; 3 Department of Nursing, Yeoju Institute of Technology, Yeoju-si, Gyeonggi-do, Korea; The University of the West Indies, JAMAICA

## Abstract

Self-care behavior plays a pivotal role in the management of chronic kidney disease. Improved self-care behavior in patients with chronic kidney disease is a key factor in health management and treatment adherence. This study aimed to evaluate the participants’ general and medical condition-related characteristics, physiological indices and the level of health literacy affecting self-care behavior in patients with chronic kidney disease in South Korea. The data of 278 participants were analyzed using t-test, analysis of variance, correlation coefficient, and linear multiple regression analysis. There were significant differences in self-care behavior scores depending on participants’ age and cohabitation status, employment, and smoking status as well as having dialysis due to end-stage kidney disease; number of comorbidities; levels of serum hemoglobin, calcium, and creatinine; and estimated glomerular filtration rate. The results of regression analysis revealed that not currently working, non-smoker, end-stage kidney disease, and positive response to the “actively managing my health” scale of the Health Literacy Questionnaire significantly affected self-care behavior in patients with chronic kidney disease, and the explanatory power of the model was 32.7%. Therefore, it is necessary to identify each patient’s barriers or needs according to individual characteristics, such as age, cohabitation and employment status, and daily life circumstances, including smoking habits, comorbidities, social support, and level of health literacy to develop efficient support strategies for promoting adequate self-care behavior with CKD.

## Introduction

The burden of chronic kidney disease (CKD) has been rising, and this trend is projected to continue due to an increase in the number of cases of diabetes and hypertension as well as an aging population in Korea. The prevalence of CKD in the US is estimated to be approximately 13% [1], and 11.4% in the population aged 30 years or older in Korea [2]. The number of patients on dialysis has tripled over 10 years [3], and is associated with high medical costs [4].

CKD is an irreversible disease and the primary treatment goal is delaying the kidney function deterioration. Integrated management involving various strategies such as monitoring of blood pressure and blood glucose, dietary restrictions, and medication adherence as well as treatment of causative diseases can improve the prognosis of patients with CKD [5, 6]. The CKD guidelines especially emphasize the importance of continuous self-care behavior and the lifestyle modification. In other words, to successfully slow CKD progression, patients must take an active role in the self-care strategy [4].

Diet is one of the main management methods for alleviating uremia, metabolic abnormalities and deterioration of renal function, and personalized diet prescriptions are provided to help the patients in maintaining proper nutritional status according to their symptoms. It is necessary to include information on forming routines foe healthy diet habits during patient education sessions, and healthcare providers must pay attention to and continuously encourage the patients with low levels of understanding of their disease [7].

Physical activity is also associated with improved cardiovascular function in patients with CKD, whereas smoking [2, 6] and a high body mass index [8] is associated with an increased risk of CKD deterioration; therefore, regular physical activity and smoking cessation are helpful. The CKD management guidelines recommend weight loss in obese adults [6]. Maintaining a normal body mass index between 18.5 and 22.9 kg/m^2^ can successfully reduce the occurrence of proteinuria [8]. As such, the integral role of self-care in CKD management is well known, but there has been limited insight into its level of engagement in all stages of CKD regardless of dialysis treatment [9].

According to previous studies, self-care behaviors and disease awareness in patients with CKD are closely related [4, 10, 11]. Thus, health literacy may be particularly crucial for patients with CKD due to the complexity of self-care needed to reduce complications such as metabolic and cardiovascular diseases. Furthermore, it has been recognized to affect the ability of patients to take action and navigate within their social networks and health systems [10]. Inadequate self-care behaviors are a result of poor disease information, which can also make it challenging to share decision-making for the therapeutic strategy [4, 7]. Interestingly, higher levels of health literacy in patients with kidney disease trended with more self-care behaviors, such as smoking avoidance, medication adherence, and diabetes management, but not with dietary restrictions and physical activity. However, the underlying reasons are not well understood [10]. A greater understanding of the patient’s everyday lived experiences is necessary for improving CKD self-care behaviors and creating efficient support strategies [12].

Hence, this study aimed to identify the medical condition-related characteristics, physiological indicators, and health literacy levels of patients with CKD that affect their self-care behavior and to provide evidence that can help these patients enhance their health status.

## Materials and methods

### Study design and participants

This cross-sectional descriptive study examined the relationship between characteristics, physiological indices, health literacy, and self-care behavior in patients with CKD.

The participants of this study were adults aged 18 years or older who were diagnosed with CKD stages 1–5, understood the purpose of this study, agreed to participate in surveys, and gave permission to access their electronic medical records. The minimum number of participants required for this study was calculated to be 245 individuals, with an effect size of .15, significance level of ⍺ = .05, power (1-β) = .95, and 26 predictors (7 sociodemographic characteristics, 10 medical condition-related characteristics, 9 health literacy factors) for linear multiple regression analysis using the G*Power 3.1.6 program. Data were collected from 285 participants. After excluding the data of seven participants who submitted incomplete questionnaires, the data of 278 were finally analyzed.

### Measurement

#### Participants’ characteristics

We investigated the general characteristics (including sex, age, education level, living status, employment status, smoking status, and body mass index) and medical condition-related characteristics (including stages of kidney disease, duration of CKD diagnosis, causes of CKD, physiological indices, current medications, and comorbidities) using surveys and the respective electronic medical records. Comorbidity was measured using the Davies Comorbidity Index (DCI) [13]. The DCI was originally developed to predict the risk of hospitalization and mortality in patients with CKD based on the presence or absence of seven comorbidities: active cancer; ischemic heart disease; peripheral vascular disease; left ventricular dysfunction; diabetes mellitus; systemic collagen vascular disease; and other significant pathologies such as asthma, cirrhosis, and chronic obstructive lung disease. DCI is scored as follows: 0 for no comorbidity, one point for one or two comorbidities, and two points for three or more comorbidities. For the physiological indices, the Kidney Disease Outcomes Quality Initiative Clinical Practice Guideline of the 2015 National Kidney Foundation, suggested that the physiological indices affecting self-care include anemia, malnutrition, and bone metabolism abnormalities [14]. Accordingly, we collected data on serum levels of hemoglobin, calcium, phosphate, and total protein; Kt/V (dialysis efficacy); blood urea nitrogen and creatinine levels; and glomerular filtration rate that were evaluated every month for patients with CKD during their regular hospital visits.

#### Health literacy

The Health Literacy Questionnaire (HLQ) is a self-reported measurement tool with 44 questions under the following 9 scales [15]: (1) feeling understood and supported by health care providers, (2) have sufficient information to manage my health, (3) actively managing my health, (4) social support for health, (5) appraisal of health information, (6) ability to actively engage with health care providers, (7) navigating the health care system, (8) ability to find good health information, and (9) understand health information well enough to know what to do. The responses to the HLQ scales 1–5 are rated using a four-point Likert scale: “strongly agree” (four points), “agree” (three points), “disagree” (two points), and “strongly disagree” (one point). The difficulty of each task in the HLQ scales 6–9 is rated with a range of one to five points, namely, “cannot do or always difficult” (one point), “usually difficult” (two points), “sometimes difficult” (three points), “usually easy” (four points), and “always easy” (five points). The Cronbach’s α values during tool development were .77-.90 and .84-.91 in this study.

#### Self-care behavior

The Chronic Kidney Disease Self-Care (CKDSC) scale was developed to measure self-care behavior in CKD patients [16]. The CKDSC-K scale consists of adherence to medication (four items), diet control (four items), exercise (three items), smoking behavior (two items), and blood pressure monitoring (two items), with responses ranging from “never” (one point) to “always” (five points) scored on a five-point Likert scale [17]. The reported Cronbach’s α values were .81–.83 in previous studies [16, 17] and 9.81 in this study.

### Ethical considerations

Data collection was conducted after obtaining approval from the Institutional Review Board (no. 1041078-202006-HR-162-01) at Chung-Ang University. The purpose and procedure of this study were explained to the participants and written consent was obtained. They were assured that there would be no disadvantages if they did not participate in this study, and that only voluntary participation was allowed. The patient medical registration numbers for checking the respective electronic medical records were written on separate papers. Immediately after completing the questionnaires and collecting data from the electronic medical records, the papers with the patient medical registration numbers were shredded. No personally identifiable information was collected.

### Data collection

The data collection period was from July 25, 2020 to December 2, 2020. The participants were recruited after obtaining permission from the concerned department head at a university affiliated hospital located in D City, South Korea. Recruitment notices were posted in front of the dialysis room and nephrology outpatient clinic at the hospital. Data collection was conducted in the dialysis room and waiting room of the outpatient clinic by two nurses who were research assistants.

### Data analysis

Data analysis was performed using SPSS Statistics for Windows, version 26.0 (SPSS Inc., Chicago, Ill., USA). The general and medical condition-related characteristics of the participants were analyzed using descriptive statistics. The differences in self-care behavior and health literacy according to the participants’ characteristics were analyzed using t-test and one-way analysis of variance. A post-hoc test was performed with an equivalence test followed by Scheffé test or Dunnett T_3_. The correlation between health literacy and self-care behavior of the participants was determined using calculated Pearson’s correlation coefficients. A linear multiple regression analysis was performed to identify factors affecting self-care behavior.

## Results

### Participants’ characteristics

The study sample included 152 men (54.7%). The mean age was 57.04 years, and 222 (79.9%) participants were cohabitating, 106 (38.2%) participants were unemployed, and 28 (10.1%) were smokers. In terms of medical condition-related characteristics, 184 (66.2%) patients were on dialysis because of end-stage kidney disease, 69 (24.8%) had CKD for <1 year, and 107 (38.5%) had CKD for ≥5 years. The most common cause of CKD was diabetes mellitus, followed by kidney disease and hypertension. In addition, 134 (48.2%) patients were taking less than four prescribed medications, and 207 (74.4%) participants had one or two comorbidities. The serum test results showed that 130 patients (47.3%) had normal hemoglobin levels, 151 (54.7%) had normal calcium levels, 122 (44.2%) had normal phosphate levels, 211 (76.7%) had normal total protein levels, 60 (32.3%) had normal blood urea nitrogen levels, 59 (21.4%) had normal creatinine levels, 47 (17.0%) had normal glomerular filtration rate levels, and 133 (89.9%) had normal Kt/V ratios (Table 1).

**Table 1:** Participants’ characteristics (N = 278).

Characteristics	Categories	n (%) or M^a^±SD^b^
General characteristics		
Sex	Male	152(54.7)
	Female	126(45.3)
Age (year)		57.04±15.22
Level of education	≤High school	189(68.0)
	≥College	89(32.0)
Living status	With family or someone	222(79.9)
	Alone	56(20.1)
Currently working	Yes	106(38.2)
	No	172(61.8)
Current smoker	Yes	28(10.1)
	No	250(89.9)
Body mass index (kg/m^2^)	<18.5 underweight	16(5.8)
	18.5–22.9 normal	102(36.8)
	≥23.0 obese	159(57.4)
Medical condition-related characteristics		
Stages of kidney disease	CKD^c^	94(33.8)
	ESKD^d^	184(66.2)
Duration of CKD diagnosis	<1	69(24.8)
	1–<5	102(36.7)
	≥5	107(38.5)
Causes of CKD (Multiple responses) (n = 289)	Diabetes	95(32.9)
	Kidney disease	60(20.7)
	Hypertension	54(18.7)
	Other	80(27.7)
Number of prescribed medications	0–4	75(27.0)
	5–9	134(48.2)
	≥10	69(24.8)
DCI^e^ scores	0	35(12.6)
	1	207(74.4)
	≥2	36(13.0)
Serum hemoglobin	≥11 g/dl (within range)	130(47.3)
	<11 g/dl	145(52.7)
Serum calcium	8.5–10.2 mg/dl (within range)	151(54.7)
	<8.5 or >10.2 mg/dl	125(45.3)
Serum phosphate	3.5–5.5 g/dl (within range)	122(44.2)
	<3.5 or >5.5 g/dl	154(55.8)
Serum total protein	6.4–8.3 g/dl (within range)	211(76.7)
	<6.4 or >8.3 g/dl	64(23.3)
Serum BUN^f^ (n = 186)	8.0–23.0 mg/dl (within range)	60(32.3)
	>23 g/dl	126(67.7)
Serum creatinine	0.5–1.3 mg/dl (within range)	59(21.4)
	>1.3 mg/dl	217(78.6)
Serum eGFR^g^	≥60 ml/min/1.73m^2^ (within range)	47(17.0)
	<60 ml/min/1.73m^2^	229(83.0)
Serum Kt/V^h^ (n = 148)	≥1.2 (within range)	133(89.9)
	<1.2	15(10.1)

^a^M = mean.

^b^SD = standard deviation.

^c^CKD: chronic kidney disease.

^d^ESKD: end stage kidney disease.

^e^DCI: Davies comorbidity index.

^f^BUN: blood urea nitrogen.

^g^eGFR: estimated glomerular filtration rate.

^h^Per hemodialysis.

### CKDSC and HLQ scores

The overall mean CKDSC score was 3.53±0.61. The mean scores for each scale were as follows: 4.59±0.54 for medication adherence, 4.06±1.04 for smoking behavior, 3.31±1.29 for blood pressure monitoring, 2.86±0.97 for diet control, and 2.81±1.18 for exercise. The HLQ scores are shown in Table 2.

**Table 2:** CKDSC^a^ and HLQ^b^ scores (N = 278).

Variables	Subscales	M±SD	Score range
CKDSC	Medication adherence	4.59±0.54	1–5
	Diet control	2.86±0.97	
	Exercise	2.81±1.18	
	Smoking behaviors	4.06±1.04	
	BP monitoring	3.31±1.29	
	Overall	3.53±0.61	
HLQ	1. Feeling understood and supported by healthcare providers	2.80±0.55	1–4
	2. Have sufficient information to manage my health	2.46±0.50	
	3. Actively managing my health	2.62±0.55	
	4. Social support for health	2.97±0.45	
	5. Appraisal of health information	2.64±0.52	
	6. Ability to actively engage with healthcare providers	3.27±0.76	1–5
	7. Navigating the healthcare system	3.10±0.80	
	8. Ability to find good health information	3.00±0.86	
	9. Understand health information well enough to know what to do	3.26±0.75	

^a^CKDSC = chronic kidney disease self-care.

^b^HLQ = health literacy questionnaire.

### CKDSC scores according to participants’ characteristics

The overall mean CKDSC scores were significantly high for those aged 65 years or older (t = -4.29, *p* < .001), those who were cohabiting (t = 3.09, *p* = .002), those currently unemployed (t = -4.28, *p* < .001), and non-smokers (t = -3.63, *p* < .001). The mean CKDSC scores for the medical condition-related characteristics were significantly high for patients with end-stage kidney disease; those with a DCI score ≥2 compared to those with a DCI score of 0; and those with abnormal levels of hemoglobin, calcium, creatinine, and eGFR (Table 3).

**Table 3:** Association between CKDSC^a^ scores and participants’ characteristics (N = 278).

Characteristics	Categories	n (%)	CKDSC	t or F (*p)*
Sex	Male	152 (54.7)	3.51±0.62	-0.51 (.610)
	Female	126 (45.3)	3.55±0.59	
Age (year)	<65 years	189 (68.0)	3.43±0.57	-4.29 (< .001)
	≥65 years	89 (32.0)	3.75±0.63	
Level of education	≤High school	189 (71.0)	3.52±0.62	-0.37 (.715)
	≥College	89 (33.0)	3.55±0.59	
Living status	With family or someone	222 (79.9)	3.59±0.61	-3.09 (.002)
	Alone	56 (20.1)	3.31±0.54	
Currently working	Yes	106 (38.1)	3.34±0.54	-4.28 (< .001)
	No	172 (61.9)	3.65±0.62	
Current smoker	Yes	28 (10.1)	3.15±0.56	-3.63 (< .001)
	No	250 (89.9)	3.58±0.60	
Body mass index (kg/m^2^)	<18.5	16 (5.8)	3.41±0.77	0.35 (.702)
	18.5–22.9	102 (36.8)	3.55±0.61	
	≥23.0	159 (57.4)	3.54±0.59	
Medical condition-related characteristics				
Stages of kidney disease	CKD^b^	94(33.8)	3.27±0.52	-5.43 (< .001)
	ESKD^c^	184(66.2)	3.67±0.61	
Duration of CKD diagnosis	<1	69 (24.8)	3.42±0.57	1.74 (.178)
	1–<5	102 (36.7)	3.59±0.65	
	≥5	107 (38.5)	3.55±0.58	
Number of prescribed medications	0–4	75 (27.0)	3.44±0.52	2.22 (.111)
	5–9	134 (48.2)	3.52±0.61	
	≥10	69 (24.8)	3.65±0.67	
DCI^d^ scores	0	35 (12.6)	3.29±0.50	5.10 (.007) a<c
	1	207 (74.4)	3.54±0.61	
	≥2	36 (13.0)	3.74±0.65	
Serum hemoglobin	≥11 g/dl (within range)	130 (47.3)	3.38±0.60	-4.16 (< .001)
	<11 g/dl	145 (52.7)	3.68±0.59	
Serum calcium	8.5–10.2 mg/dl (within range)	151 (54.7)	3.46±0.54	-2.28 (.023)
	<8.5 or >10.2 mg/dl	125 (45.3)	3.63±0.67	
Serum phosphate	3.5–5.5 g/dl (within range)	122 (44.2)	3.52±0.63	-0.49 (.622)
	<3.5 or >5.5 g/dl	154 (55.8)	3.55±0.59	
Serum total protein	6.4–8.3 g/dl (within range)	211 (76.7)	3.54±0.62	0.391 (.696)
	<6.4 or >8.3 g/dl	64 (23.3)	3.51±0.59	
Serum BUN^e^ (n = 186)	8.0–23.0 mg/dl (within range)	60 (32.3)	3.35±0.50	-1.33 (.187)
	>23 g/dl	126 (67.7)	3.47±0.57	
Serum creatinine	0.5–1.3 mg/dl (within range)	59 (21.4)	3.34±0.52	-2.90 (.004)
	>1.3 mg/dl	217 (78.6)	3.59±0.62	
Serum eGFR^f^	≥60 ml/min/1.73m^2^ (within range)	47 (17.0)	3.35±0.52	-2.39 (.018)
	<60 ml/min/1.73m^2^	229 (83.0)	3.58±0.62	
Serum Kt/V^g^ (n = 148)	≥1.2 (within range)	133 (89.9)	3.71±0.62	-0.42 (.673)
	<1.2	15 (10.1)	3.79±0.72	

^a^CKDSC = chronic kidney disease self-care.

^b^CKD: chronic kidney disease.

^c^ESKD: end stage kidney disease.

^d^DCI: Davies comorbidity index.

^e^BUN: blood urea nitrogen.

^f^eGFR: estimated glomerular filtration rate.

^g^Per hemodialysis.

### Correlations among measurement variables

The results of the correlation analysis of the association between the nine scales of the HLQ and the CKDSC score are shown in Table 4. The correlation coefficient for the association between the CKDSC score and the score on scale 3 (“actively managing my health”) was the highest (*r* = .46), and the correlation coefficient with the score on the scale 8 (“ability to find good information”) (*r* = .16) was the lowest (Table 4).

**Table 4:** Correlations between nine scales of HLQ^a^ and CKDSC^b^ (N = 278).

Scales	HLQ1. Feeling understood and supported by healthcare providers	HLQ2. Have sufficient information to manage my health	HLQ3. Actively managing my health	HLQ4. Social support for health	HLQ5. Appraisal of health information	HLQ6. Ability to actively engage with healthcare providers	HLQ7. Navigating the healthcare system	HLQ8. Ability to find good health information	HLQ9. Understand health information well enough to know what to do
	*r* (*p*)								
HLQ2	.46 (< .001)								
HLQ3	.45 (< .001)	.65 (< .001)							
HLQ4	.54 (< .001)	.43 (< .001)	.43 (< .001)						
HLQ5	.39 (< .001)	.71 (< .001)	.68 (< .001)	.32 (< .001)					
HLQ6	.38 (< .001)	.44 (< .001)	.35 (< .001)	.41 (< .001)	.30 (< .001)				
HLQ7	.23 (< .001)	.51 (< .001)	.30 (< .001)	.33 (< .001)	.42 (< .001)	.77 (< .001)			
HLQ8	.12 (.053)	.51 (< .001)	.29 (< .001)	.24 (< .001)	.46 (< .001)	.67 (< .001)	.85 (< .001)		
HLQ9	.17 (.005)	.43 (< .001)	.33 (< .001)	.28 (< .001)	.38 (< .001)	.77 (< .001)	.81 (< .001)	.80 (< .001)	
CKDSC	.33 (< .001)	.28 (< .001)	.46 (< .001)	.28 (< .001)	.27 (< .001)	.28 (< .001)	.19 (.002)	.16 (.009)	.20 (.001)

^a^HLQ = health literacy questionnaire.

^b^CKDSC = chronic kidney disease self-care.

### Factors affecting CKDSC

The model derived by analyzing the effect of the participant’s characteristics and health literacy on CKDSC using a linear multiple regression analysis was significant, and there was no multicollinearity (Durbin–Watson statistic, 1.93, tolerance, .18–.93, variance inflation factor, 1.08–5.52). The variables of current unemployed status (B = .16), non-smoker status (B = .32), end-stage kidney disease (B = .31), and the “actively managing my health” scale (B = .41) significantly affected the CKDSC score, and their explanatory power was 32.7%. The variable “age 65 years or older” (B = .14), although not significantly affected by CKDSC, was near the significant level (*p* = .059) (Table 5).

**Table 5:** Factors associated with participants’ self-care behaviors (N = 278).

Characteristics	Categories	B (SE^a^)	*p*
	Constant	1.46 (.354)	< .001
Age (year)	≥65 years	.14 (.075)	.059
Living status	With family or someone	.04 (.086)	.644
Currently working	No	.16 (.076)	.037
Current smoker	No	.32 (.103)	.002
DCI^b^ scores	0	-.03 (.193)	.896
	1	-.07 (.095)	.450
Stages of kidney disease	ESKD^c^	.31 (107)	.004
Serum hemoglobin	≥11 g/dl (within range)	-.06 (.074)	.426
Serum calcium	<8.5 or >10.2 mg/dl	-.03 (.072)	.691
Serum creatinine	>1.3 mg/dl	.06 (.154)	.717
Serum eGFR^d^	≥60 ml/min/1.73m^2^ (within range)	.16 (.185)	.382
HLQ^e^1. Feeling understood and supported by healthcare providers		.03 (.079)	.716
HLQ2. Have sufficient information to manage my health		-.15 (.103)	.138
HLQ3. Actively managing my health		.41 (.090)	< .001
HLQ4. Social support for health		.08 (.090)	.353
HLQ5. Appraisal of health information		-.02 (.101)	.859
HLQ6. Ability to actively engage with healthcare providers		.07 (.076)	.377
HLQ7. Navigating the healthcare system		-.02 (.088)	.795
HLQ8. Ability to find good health information		.10 (.082)	.246
HLQ9. Understand health information well enough to know what to do		-.01 (.083)	.871
F-value (*p*)		7.65 (< .001)	
R^2^		.38	
Adjusted R^2^		.33	

^a^SE = standard error.

^b^DCI: Davies comorbidity index.

^c^ESKD = end stage kidney disease.

^d^eGFR = estimated glomerular filtration rate.

^e^HLQ = health literacy questionnaire.

## Discussion

The mean CKDSC score in this study was 3.53 points, which is similar to that reported by Wang et al. [18] in their study on self-care in patients with CKD in Taiwan measured using the same tool (3.65). However, their scores for diet control (2.86 vs. 3.56) and exercise (2.81 vs. 3.14) scales were somewhat lower than our scores [18]. Participants’ CKDSC scores were significantly higher for those aged 65 years or older, those who were cohabitating, and those who were currently unemployed, which was consistent with the results of previous studies [4, 18]. Compared with those living alone, people living with a cohabitant helped them practice healthy lifestyles [19]. It may be difficult for employed people to practice self-care owing to their work schedule, stress, and lack of time [4, 10]. Because the environments and needs of patients with CKD are different, medical professionals should make efforts to identify their needs and provide customized care to help them manage chronic diseases [20]. It is necessary to identify patients with CKD living alone and in need of social support and to invigorate support systems through CKD patient self-help groups and social groups that can help them practice health management [21]. In addition, the use of a smartphone or remote system can help increase patient access to services involving dietary and physical activity monitoring and enhancement.

The results of our analysis of self-care behavior scores according to medical condition revealed that the self-care behavior score was high for those with low hemoglobin level, high creatinine level, low eGFR, and many comorbidities with regard to the physiological indices. The self-care behavior score was higher in patients with end-stage kidney disease who were on dialysis when compared to other patients with CKD. These results are consistent with the results of Wang et al. [18], who reported that the overall mean CKDSC score was higher for patients with late-stage CKD than for those with early stage CKD [18] as well as of Tsai et al. [4], who showed that low serum hemoglobin level was associated with high self-care behavior [4]. The low self-care scores of patients with early stages of CKD may be because they do not yet present with any physical symptoms owing to minor kidney damage, and thus lack a sense of their disease and do not realize the importance of lifestyle habits such as diet control and exercise. Meanwhile, patients with dialysis-dependent CKD may have already progressed to end-stage kidney disease with complete loss of kidney function, resulting in a decrease in eGFR and increase in creatinine levels. Because of the deficiency of renal erythropoietin production, the production of red blood cells in the bone marrow decreases, resulting in anemia and gradual development of physical symptoms due to uremia [18]. Therefore, patients with end-stage kidney disease tend to have higher awareness of the importance of routine CKD management and higher self-care behavior scores than patients with early stages of CKD. Comorbidities such as hypertension, diabetes, anemia, and obesity exacerbate renal disease, and the resulting renal function decline may lead again to the exacerbation of existing comorbidities [22]. As patients with CKD experience a disease continuum, they need to continue to practice self-care behaviors [4].

RobatSarpooshi et al. [23], who investigated the correlation between health literacy and self-care behavior in patients with chronic diseases such as diabetes, also reported that health literacy was highly related to self-care behavior. Because steady management and treatment of chronic diseases such as CKD are recognized as vital factors, active intervention and patient education by nurses as well as support from people around may be closely related to self-care behavior. In a systematic review on information important to CKD patients, the demand for practical information was high among long-term CKD patients, and they frequently pursued patient education rather than seeking new medical resources [24]. This could be explained by the low correlations of scale 7 of the HLQ (“navigating the healthcare system”) and scale 8 of the HLQ (“ability to find good health information”) with CKDSC in the current study.

We found that scale 3 of the HLQ (“actively managing my health”) positively affected CKDSC. As “actively managing my health” refers to the act of recognizing the importance of one’s own health and making health-related decisions with a sense of responsibility [15], this might have the strongest effect on self-care behavior. Pre-dialysis stage had more negative effects on self-care than dialysis. This may be because the number of hospital visits and how a trusting relationship is established between the patient and their healthcare providers differ according to dialysis/non-dialysis status and the type of treatment [21, 25]. In South Korea, patients with pre-dialysis CKD visit an outpatient clinic every one to three months for their kidney disease status to be examined. Meanwhile, patients on peritoneal dialysis not only visit an outpatient clinic once a month, but they also have access to telephone consultations from nurses as needed. Patients on hemodialysis visit a hospital three times a week to receive 3–4-hour-long dialysis treatments and consultation from nurses during their dialysis treatment. These consultations may be related to inadequate engagement in self-care behaviors, such as dietary restrictions, exercise and medication adherence, and the management of comorbidities and complications. Patients with dialysis-dependent CKD are more likely to feel supported by their healthcare providers, which causes improvement in their self-care behavior.

The results of this study showed that health literacy, except for the scale 3 of the HLQ (“actively managing my health”), had no significant effect on self-care behavior in patients with CKD. Although health literacy is an important element for practicing self-care in patients with CKD [7], it can be insufficient to improve self-care behavior, and the possibility that other factors may have a greater effect on self-care behavior in patients with CKD should be considered. In other words, self-care behavior in patients with CKD are complex as individuals may have different perspectives on their illness, and their priorities, expectations for support, trust in healthcare providers and the healthcare system, and how they go about carrying-out their self-care may vary. Additionally, studies conducted up to now have investigated health literacy in CKD using tools for measuring only the functional aspects of health literacy, such as numeracy skills and reading comprehension [26–28], and have not captured health literacy scales that are important in healthcare delivery, such as the ability to appraise health information or engagement with healthcare providers [29]. Given that only a limited number of studies have comprehensively identified challenges that patients with CKD patients experience in understanding and applying health information in their daily life, further studies are needed in this context.

Finally, this study has a few limitations. As self-care behavior and health literacy in patients with CKD patients were measured using a self-report questionnaire, the participants’ recall bias might have affected the results of this study. We recruited patients with CKD at a single hospital, which may limit the generalizability of results. The study had slightly higher proportion (66.2%) of participants who were on dialysis; therefore, care should be taken when interpreting the results. Considering that various factors are included in the self-care behavior of patients with CKD, including dietary restrictions and disease management, this study is important because to our knowledge, this is the first study to analyze the effects of health literacy on self-care behavior in Korean patients with CKD using a tool that encompassed a multidimensional concept of health literacy including access to healthcare, interactions with experts, and social support systems instead of using a tool that measured only functional health literacy, such as reading and writing abilities in patients with CKD.

## Conclusion

This study analyzed factors affecting self-care in patients with CKD using a questionnaire survey and medical record analysis. The results indicate the importance of having a responsible attitude toward actively managing one’s health. Specialized interventions are needed to help patients with CKD who have a job, are living alone, and have a low frequency of contact with nurses or healthcare providers. Further multi-site studies on the relationship between health literacy and self-care according to CKD stage are needed to acquire basic data that can help improve self-care behavior in patients with CKD. In addition, to enhance self-care behavior in patients with CKD, future studies can investigate the effects of the use of Korean healthcare systems on applying health information related to CKD.

## Supporting information

S1 Dataset (XLSX)
